# ‘It's a double whammy’: A qualitative study of illness uncertainty in individuals with Parkinson's disease in the context of COVID-19

**DOI:** 10.1177/17423953211043101

**Published:** 2022-12

**Authors:** Jane Simpson, Nicolò Zarotti, Sandra Varey, Eleftherios Anestis, Carol Holland, Craig Murray, Fiona JR Eccles

**Affiliations:** 83563 Division of Health Research, 151268 Faculty of Health and Medicine, Lancaster University, UK

**Keywords:** Parkinson’s disease, COVID-19, qualitative, illness uncertainty

## Abstract

**Objectives:**

The purpose of this study was to explore the experiences of individuals with Parkinson's through the theoretical lens of illness uncertainty during the first UK full lockdown period (March–June 2020) put in place due outbreak of the COVID-19 pandemic.

**Methods:**

Individual semi-structured interviews were carried out via telephone in May 2020 with 10 individuals with Parkinson's (six men and four women) recruited from Parkinson's UK. Interviews were recorded and transcribed verbatim, and thematic analysis was adopted to analyse the resulting data.

**Results:**

Four overarching themes emerged from the interview data: (1) COVID-19 amplifying existing fears and difficulties around the uncertainty of Parkinson's; (2) practical and psychological efforts to manage uncertainty; (3) benefit-finding as a way of acknowledging the positives of lockdown; (4) risk and future management in the context of uncertainty.

**Discussion:**

Participants reported a range of implicit and explicit strategies to cope with the ‘double whammy’ of uncertainty caused by having Parkinson's during a global pandemic. While these were generally successful in maintaining well-being, it is important that such successful accounts are used to help inform novel strategies and interventions targeting individuals who might need additional support.

## Introduction

Chronic illnesses often cause uncertainty.^1,2^ Mishel's theory^1^ – reformulated specifically for chronic conditions^3^ – represents one of the most influential works on illness uncertainty, defining it as ‘the inability to determine the meaning of illness-related events [that] occur in situations where the decision-maker is unable to assign definite values to objects and events and/or is unable to accurately predict outcomes because sufficient cues are lacking’^3^ (p. 256). This highlights four factors that tend to form part of the illness uncertainty experience: ambiguity about the state of the illness, complexity regarding treatment and healthcare systems, lack of information about diagnosis and seriousness and unpredictability about course and prognosis.^4^ While uncertainty can be associated with positive outcomes,^4^ it is more commonly experienced negatively – not only as psychologically uncomfortable, but related to lower psychological well-being, increased anxiety, depression, anger, illness intrusiveness, and decreased hope and problem-focused coping responses.^5,6^ Given the difficulty/impossibility of eliminating uncertainty in unpredictable chronic conditions, the optimal management of uncertainty has become paramount.^7^

Illness uncertainty has also been theorised in relation to coping. As part of Lazarus and Folkman's theory of stress and coping,^8^ feelings of uncertainty during the primary appraisal of illness manageability may act as a cognitive stressor and increase the perceptions of unmanageability.^9^ This then influences secondary appraisal – for example, individuals’ personal, systemic and societal resources to manage the challenges of the illness. Where these are also characterised by uncertainty, distress may increase and affect attempts to manage that additional uncertainty.

One condition where uncertainty features prominently is Parkinson's disease,^1^ a neurodegenerative condition with no cure.^10^ Traditionally considered a movement disorder, Parkinson's is also associated with several psychological difficulties.^11^ Its illness trajectory is unpredictable and response to symptomatic treatment may also become increasingly variable as the disease progresses.^12,13^ The path to diagnosis can be fraught, and effective healthcare communication is often reported as lacking.^14,15^

Considering the overlap between the experiences of people with Parkinson's (PwP) and Mishel's theory of illness uncertainty, it is not surprising that high levels of uncertainty have been well established in this population,^16^ including among those with younger onset (i.e. before age 50 years^17^). Moreover, PwP are also generally reported to experience uncertainty negatively,^12,18^ especially due to lack of clarity about the nature of symptoms (e.g. Parkinson's or normal ageing), concerns about the unpredictability of the future, and consequent avoidance of long-term planning.^19^ In turn, a number of maladaptive strategies have been argued to result, at least in part, from this uncertainty, including excessive behavioural responses.^20^ Higher levels of uncertainty in PwP have also been associated with increased disease severity, lower perceived social support and decreased resilience.^16^ However, a number of informal strategies have been reported to reduce uncertainty, such as focused information-gathering and increasing perceived control,^21^ which could benefit from individual approaches to care – for example, allowing each patient to decide the amount of information they need at any stage.^22^

Since January 2020, the threat of the COVID-19 pandemic has led to unprecedented responses globally.^23^ Even in countries with a certain confidence in healthcare systems, wider support structures, and economic stability, this has been progressively characterised by a degree of uncertainty much higher than in other recent pandemics.^24^ However, although people with chronic illness may be more at risk of psychological difficulties in the current global situation,^25^ very little is known about the experiences of individuals with neurodegenerative diseases such as Parkinson's during the pandemic, especially in terms of mental health. Moreover, although quantitative research has identified uncertainty as important, little qualitative research exists regarding this in Parkinson's. As the pandemic presents a set of circumstances likely to exacerbate Parkinson's-related difficulties, this study offers an opportunity to provide a more in-depth understanding of uncertainty, its exacerbation, and required behavioural changes in response to COVID-19 in PwP.

## Method

### Design

This study examined a subset of the data collected as part of a longer-term, qualitative examination of the COVID-19 outbreak for PwP. The main aim of the qualitative component of this project focused on exploring each participant's daily life, health status and activity levels before COVID-19, understanding their experiences of social distancing/self-isolation and any impact on their lives and wellbeing. The methodological approach was one of pluralism – allowing different analyses to be conducted in order to produce ‘multiple, complex and varied understandings of phenomena’,^26^ (p. 182). The present study explored the data from the first set of interviews to understand illness uncertainty in PwP in the context of COVID-19 using thematic analysis, a flexible method which can accommodate both inductive and deductive approaches.^27^ Our own approach sits in between these two poles: while the data have not been collected with the construct of uncertainty in mind, the analysis drew upon theory and empirical findings regarding illness uncertainty to identify data which illuminate these particular aspects of participants’ experiences. Similarly, neither the original protocol nor questions focused on experiences of uncertainty, meaning that, if participants raised these issues, they were doing so spontaneously reflecting their own concerns and priorities.

#### Participants

Ten PwP (six men and four women) were interviewed. The mean age was 63.8 years, with an average of 8 years since diagnosis (Table 1). All participants were recruited online via Parkinson's UK, the UK's largest charity dedicated to the condition. Most participants were managing at home without additional help, with only one more advanced-stage patient having 24-h live-in care since the start of the pandemic. The sample was relatively independent, functioning well, and with no reported signs of severe psychological distress at the time of interview.

**Table 1.** Participant demographic and clinical characteristics.

P	Age	Gender	T since diagnosis	T since onset	Living status	Ethnicity	Nationality	Main reported PD symptoms
1	67	Male	13 years	13.5 years	Lives alone (carer 24/7 during lockdown)	White	British	Poor balance, Shuffling, Hallucinations
2	60	Male	5 years	6 years	Lives alone	White	British	Resting and action tremor, impaired fine motor skills
3	59	Male	3 years	5 years	Lives with wife (and daughter during lockdown)	White	British	Resting tremor
4	63	Female	5 years	6 years	Lives with husband	White	British	Rigidity, shuffling, freezing
5	66	Male	10 years	11 years	Lives with wife	White	British	Shuffling, fatigue, (difficulty walking, falls), loss of smell, memory problems
6	65	Female	8 years	12 years	Lives with husband	White	British	Tremor, falls, freezing, dyskinesia
7	62	Male	4 years	3 years	Lives alone	White	British	Loss of strength in left leg and hand, loss of synchronisation in the left leg and hand, rigidity, fatigue
8	63	Male	9 years	Not able to pinpoint	Lives with wife (and daughter during lockdown)	White	British	Posture problems, pain, shuffling
9	71	Female	5 years	7 years	Lives alone	White	British	Tremor, freezing, pain, poor movement coordination, poor blood circulation
10	62	Female	18 years	Not able to pinpoint	Lives with husband	White	British	Falls, freezing, poor movement coordination

#### Procedure

An invitation to take part in the study was sent to individuals via Parkinson's UK's mailing lists. Those who expressed interest were then sent the participant information sheet and contacted to answer any further questions. Of 12 individuals who expressed their interest, two could not participate due to not being contactable or having a diagnosis different from Parkinson's. Ethical approval was granted from the first author's academic institution and informed consent was obtained from all participants.

Semi-structured individual phone interviews were conducted in May 2020, when UK Government guidance included no social contact outside the household, cancelled or rescheduled routine appointments, and additional measures such as shielding.^28^ The interviews included open questions exploring individuals’ perceived impact of COVID-19 restrictions on their psychological well-being, social interactions, and clinical care, and were inspired by early reports on the effects of the pandemic.^29^ All interviews were conducted by two authors (JS, AE) – both doctoral-level psychologists with experience in qualitative data collection – and were digitally recorded and transcribed verbatim.

### Analysis

Data were analysed with thematic analysis, which can be used with different theoretical frameworks.^27^ In this study, themes were derived from a focus on illness uncertainty, taking a realist, phenomenological perspective – that is, a close relationship is assumed between how people think and behave, and the language they use to describe this is believed to reflect their understandings and meanings. Each transcript was read multiple times to identify relevant codes, which were grouped iteratively and formed the overarching themes. This helped summarise initial interpretations and informed the search for patterns across the dataset. Throughout the process, themes were constantly reviewed to ensure they had sufficient supporting data.

Based on Yardley's principles,^30^ the analysis was conducted by the first author and then discussed with the other authors to ensure rigour, transparency, and adherence to the data.^27^ All authors considered the findings alongside their knowledge of Parkinson's to assess the sensitivity to the context of existing research^30^ and credibility to the reader. The identified themes are described below, with verbatim quotations to enhance the transparency of the analysis.^31^

## Results

Theme 1. ‘When this came into being, it made it more of a challenge’: COVID-19 amplifying existing fears and difficulties around the uncertainty of Parkinson's.

Fears and concerns associated with managing the uncertainty of living with Parkinson's were amplified during the lockdown period, including those relating to hospitalisation, independence, identity, choice, and loss of function. Fear of hospitalisation was either based on negative expectations or actual previous experience, fuelled by specific anxieties around medications potentially not being available at the right time given the other priorities that medical staff would be facing:

P5 – The big thing with Parkinson's is you should get it [medication] on time. And the number of pills I’m taking are many and varying. I would worry […] if that was put aside cause they’re concentrating on oxygen and breathing and what have you. How would I react, how would it be? I’ve never gone without medication more than half an hour or so.

Other examples included isolation and worry around medical procedures, such as being on a ventilator, along with its negative symbolic value, representing a belief that life was potentially at its end:

P2 – If it got to that on the ventilator and then it would set off a lot of other things with your Parkinson’s, your Parkinson’s wouldn’t just go away. Me thinking about it, I’m now shaking.

Further general fears relating to the psychological implications of living with the uncertainties of Parkinson's during the epidemic also emerged. For example, for one participant COVID had resulted in switching from receiving carers’ visits for a few hours a day to having a 24-h live-in carer. While this improved safety and support, it also resulted in a loss of independence, making the participant feel ‘lunged at’ by the full-time carer, who constantly worried about him falling:

P1 –Because outside, I have no balance problems. Inside they [carers] see me wobble, they don’t know that I know I am wobbly and I’m about to correct it.

The restrictions also intensified the limits already placed on individuals with the condition:

P2 –The inability to go somewhere you wanna go, even if you didn’t wanna go, the choice is gone from you. The choices have been lessened.

Participants felt *‘as though you are prisoned’* (P2), with negative effects on identity and beliefs around their role in their domestic partnership:

P4 –I can’t go shopping on my own, which I didn’t do before, I went with my husband, but I felt I was contributing at least

Participants reported fears around the loss of independence, which were amplified due to the decision to put PwP on the ‘vulnerable’ list This curtailed social events and the option of using other contacts to take part in social events:

P8 –I’m labelled as someone with a problem, who is vulnerable. Then people are making decisions for me, and I’m losing independence even more. […] So, my wife has to take me, whereas I would usually have several other options.

Loss of individuality also appeared to have occurred for some, with the Parkinson's label now defining even more how individuals were both regarded and treated. For example, Participant 3 had been working in school and wanted to continue providing support to the children of key workers. However, he was encouraged to be ‘furloughed’:

P3 –I was made to realise that, I was sort of, not trouble, but indicated quite strongly that perhaps I shouldn’t really be volunteering, it wouldn’t be a good idea. […] I don’t know, I’m in this medium, high-risk register, but I don’t feel medium, high-risk, ‘cause I am fairly young and I have Parkinson’s’. I think Parkinson’s is in the high-risk register because it’s an older person’s disease and, obviously, the older you get, the greater the risk.

The effects of the COVID restrictions on deteriorating physical function were also a cause of concern:

P7 –I didn’t feel too bad, apart from the fact that I’ve been diagnosed with Parkinson’s, I drag my left leg a little bit when I’m walking on the flat, I’ve lost strength in the left hand, not too much wrong with me really.

Such actions led to concerns that Parkinson's symptoms would gain more prominence in any assessment of general overall physical ability and their individual ability would be overshadowed by narratives which over-emphasised the importance of their Parkinson's.

Theme 2. ‘I do think it's gonna be the next few years before we are in control’: Practical and psychological efforts to manage an uncertain situation.

All participants attempted to gain control of the uncertainty of the situation – with some attempts more successful than others – and also accepted that the uncertainty they were experiencing was additional to ‘*uncertainty of Parkinson's’*, which ‘*keeps you on your toes and you don't know what's gonna happen’* (P2). Many participants also realised that the two sources of uncertainty (Parkinson's and COVID) amplified each other, and that their approach to the additional uncertainty created by social restrictions was pragmatic and focused on acceptance:

P9 –They’re talking about opening some shops next week, but then they say you can’t touch anything, and you can’t this or that. Life is going to be quite different, but it’s going to be different for everybody, we’ve all got to cope with it and get on with it. It’s something I can’t change.

Others managed by not only accepting the situation, but also the restrictions, which seemed to provide some certainty and helped create a feeling of control:

P2 –I’m all for rules and regulations, ‘cause I like to obey by the rules and regulations. If you tell me to do something, I will do it’.

Other efforts to gain control resulted in being practical and positive, embracing new technology to facilitate social contact and being open to further learning by reading new books. Being flexible around timeframes for a resumption of ‘normality’ was also important.

P2 –I think there’s light at the end of the tunnel. Ι’ve moved my goal post now, this is to get out of the lockdown.

The importance of psychological strategies to maintain a sense of control was also emphasised. Positive comparisons of self to others were used to mitigate the effects of the restrictions:

P5 –We’ve got a large garden at the back of the house, so it doesn’t feel penned in like, those, you know, fifth floor in a high rise flat with 3 children type of things, don’t know how you’d cope.

P6 –My auntie who lives in (town), she’s in her 80 s, she has long-term health problems and she’s on her own. She’s in worse situation than us.

In terms of practical strategies, it was also important to make plans after lockdown and to have faith and hope that events would be rescheduled. Such plans were often detailed and carefully considered:

P5 –We have plans ready. […] I got a broom, I measured a brush handle, a sweeping brush handle. And if I have it out with my arm, and the handle stretched horizontally, it’s 2 metres.

Hope was clearly articulated for a return to ‘normal’, with events considered ‘*notcancelledbut postponed*’ (P10). Expectations related to the resumption of specific activities, like being able to ‘go in the same car for a day out somewhere, for a drive’ (P7), but also for a general end to the pandemic through the discovery of a vaccine:

P7 –I do think it’s gonna be the next few years before we are in control, probably. […] I think they are making promises that they can’t stick to, I don’t think the vaccine is going to be here in September like they’re saying, I think it’s twelve months away.

Participants also discussed their faith in experts and how knowledge improved their sense of control over the situation:

P7 –I got a good friend, she’s a research microbiologist and she directs me, she’s involved in all this, so I get quite a bit of inside information.

The psychological toll of dealing with COVID-19 against a backdrop of also managing a serious health condition was acknowledged by all participants. Some were also able to accept the negative feelings they occasionally felt by reframing the issue:

P2 –I say to people ‘everyone feels it’s difficult with this lockdown, but my life was difficult before this anyway, so it’s even more difficult now’. I think there’s a little bit of self-pity, but I’m entitled to that I think, sometimes.

P5 –I was due an appointment to be sent out at by the end of April. And I’m loathed to ring, possibly they’ve got enough on at the moment, to me it’s a query as opposed to a problem.

Thus, attempts to increase control and reduce uncertainty were evidently clear, and references to increasing control were frequent across all interviews.

Theme 3. ‘I feel as though everybody is in it together’: benefit-finding as a way of acknowledging the positives of lockdown.

Despite multiple challenges, participants were able to see positives in the pandemic both on an individual and societal level. These were important in balancing the potential psychological distress from having to manage an additional set of stressors, by reassessing priorities and feeling an increase in personal resilience:

P2 –It’s made me even stronger. It’s given everyone a bit of a kick up the backside. I think society in general will benefit … family and friends and loved ones are more important than anything else, and I think it might make the world a better place.

Accordingly, many accounts detailed how community cohesion and the strengthening of bonds with neighbours had increased. Activities had changed, often with the result of being more satisfying:

P3 –We’re closer to the neighbours than we ever were. […] My next-door neighbour, who is 86, we do shopping for him when he needs, I give him a phone call every day, check he’s alright, that sort of thing. So, it’s changed, some of the things we are not doing, but some things we are doing.

It was also apparent that the societal experience had also led to a sense of ‘togetherness’ which helped mitigate the feelings of ‘difference’ often reported by individuals with Parkinson's:

P2 –To some degree I feel as though everybody is in it together, the COVID, we are. So, I do feel a sense of belonging to others.

Where negatives were experienced, these were also mitigated by the sense of this being a shared community response:

P7 –I can’t say I don’t get down with it occasionally, but I think everyone is getting down.

Another positive experience was the release from some of the everyday pressures of managing the condition. While previously some aspects of medication regimes had to be carefully planned around social needs, the changes in social routine dictated by COVID-19 had brought some flexibility, which could feel liberating:

P4 –In some ways, it’s been easier. I don’t have to worry about making sure I’ve taken my tablets so that I can go to the pub on Monday night for the quiz and walking, walk in or out alright, which is my priority.

Others also appreciated some time off their social obligations, almost a *‘sabbatical from normal life’* (P10), which had a positive effect on levels of stress and well-being:

P10 –I don’t have the commitments I used to have, which used to make me stressed. I was worried that I might go off when I’m out or have a fall or whatever. I’m a lot more relaxed when I’m at the house and garden with my husband.

Thus, benefit-finding – which can be common in individuals with health conditions^32^ – had the effect of mitigating the psychological challenges and threats to positive self-identity which the participants had worked hard to develop since their diagnosis of Parkinson's.

Theme 4. ‘I like to think, if I do get it … I can throw it off’: Risk and future management in the context of uncertainty.

While some of participants’ fears related to contracting COVID-19, similar levels of anxiety were also expressed about lockdown causing permanent effects on their health and well-being, for example, due to cancelled health appointments. This led some to express considerable anxiety around risk and the future:

P6 –I’m really worried, because I don’t think this is over with. I am not being pessimistic, I’m being realistic really, because I know it can spike again and I’m really worried about that.

This also appeared to trigger a more *laissez-faire* attitude in some participants, due to the awareness of their condition and the belief that their life-expectancy was limited:

P8 –Because I’ve got such a serious problem I’m getting to the point where, you know, I wouldn’t say I’m ‘cavalier’ about it, but at the most, I have no fear. Because my life-long prospects are not that great, so I’m almost thinking ‘bugger it’.

Anxieties were also expressed around physical changes experienced during the lockdown restrictions becoming permanent, as Parkinson's was viewed as a potential impediment to re-attaining previous levels of activity:

P7 –I’ve become more dormant, more lethargic, and I’m hoping that when everything’s lifted and we go back to the walking, my health will come back to me, but with the Parkinson’s, I’m not sure about that really.

Moreover, the anxiety associated with the risk of going out after lockdown was feared to exacerbate Parkinson's symptoms even further:

P2 –My tremors cause me problems, if I went out. I’d be more anxious ‘cause of people. So, I’m not saying I wouldn’t go out, but I’d be anxious going out now because of the COVID, so I’d stop bringing attention to myself and I’d probably be shaking’.

A further challenge was represented by the difficulty in differentiating between problems exacerbated by the pandemic and the progression of Parkinson's:

P5 –Since the lockdown it’s been worse … half an hour beforehand I’m due [to take medication], I get the warning signals that I’m due and I look at my watch and ‘what’s going on? I feel bad’, but they seem to wear off more quickly […] it can’t be the virus ‘cause I haven’t got it. But my body is reacting differently … and my ‘not good’ feelings are coming more often’.

P9 –It’s easy just to sit about, watch TV, and read the paper. So, I don’t know how much of that is actually the COVID, but I do think that the Parkinson’s is worsening as well. So, unfortunately, I think I’ve got the double whammy.

Therefore, although coping strategies differed, assessing the uncertainties of the future was an explicit concern to all participants, often complicated by Parkinson's and some of the generalisations around it.

## Discussion

Four themes emerged from the present study: (1) COVID-19 amplifying existing fears and difficulties around the uncertainty of Parkinson's; (2) practical and psychological efforts to manage uncertainty; (3) benefit-finding as a way of acknowledging the positives of lockdown; (4) risk and future management in the context of uncertainty. Generally, the findings confirmed that anxiety around COVID-19 was a further source of uncertainty in addition to Parkinson's, with both psychological and somatic consequences,^33^ as well as individual differences in participants’ ability to tolerate it.^34^ These differences also resulted in different coping mechanisms, with some participants taking major steps to manage the situation (e.g. 24-h support) and others still wanting to work and be regarded as having the same level of risk as to the general population. Interestingly, the communal feeling of ‘all being in the same boat’ mitigated the stigma that can accompany some enforced health quarantines,^35^ while ‘taking control’ was seen as paramount.^28^ Since illness uncertainty has been theoretically formulated as loss of control,^36^ perceived control was unsurprisingly central in PwP's coping processes,^11^ which is also consistent with findings from other neurodegenerative conditions.^37,38^

Theme 1 shows how the ‘double whammy’ of living with Parkinson's and dealing with a global pandemic both accentuated existing physical and psychological challenges and created new ones, which in turn exacerbated the fears around living with an unpredictable chronic illness. However, participants’ narratives appeared to revolve around many of the known empirical and theoretical responses to managing uncertainty. As outlined in Theme 2, these included a broad range of attempts to regain control, including following rules/guidance, referring to experts, making detailed plans and, where control was not possible, adopting a non-critical approach to their own emotional reaction. These often resulted in participants feeling they were managing well, or as well as they could.

As shown in Theme 3, participants were also able to see the positives of the consequences of the pandemic – another indication of the wide range of strategies used to manage the uncertainties caused by the illness and amplified by the pandemic. This finding is consistent with several theoretical approaches, perhaps most notably the theory of cognitive adaptation,^39,40^ which proposes that perceiving benefits in response to a chronic stressor can be viewed as a cognitive strategy employed to mitigate the negative impact of a disease. Moreover, one of the hypothesised functions of benefit-finding is to gain mastery, seen here as an overarching attempt to maintain control in the face of an extremely challenging situation.^40^ On this basis, benefit findings may reduce adverse health outcomes by reducing distress, which may result in a number of alternative ways to maintain health and well-being.^40^ While it could be argued that benefit finding may reflect a coping strategy for negative situations in general, past evidence shows that is often adopted against illness uncertainty specifically.^32^ As a consequence, PwP may have adapted it here to cope with the challenges of the pandemic.

Theme 4 delineated how participants decided to manage risk and the future in the context of personal and global uncertainties. For managing uncertainty in chronic illness specifically, Mishel^3^ suggests that growth and adapting to a new value system are important goals, achievable through the four stages of managing uncertainty: (1) understanding the causes of uncertainty; (2) perceiving uncertainty as a threat or opportunity; (3) attempts to manage or maintain the uncertainty according to whether it is a threat or opportunity; (4) the state of adaptation that results from implementing coping efforts. This study has provided evidence consistent with this formulation, in that the themes highlighted that PwP understood the heterogeneous causes of their uncertainty during pandemic, were able to perceive uncertainties as both threats (e.g. hospitalisation) and opportunities (e.g. increased connection), and respond accordingly. For most participants, this highlighted relevant coping skills, acknowledging personal growth and adapting to a new way of being, albeit with hope for return ‘normality’ in the future. The timescale for this development was relatively short – only 4 months from the announcement of the pandemic to the interviews – which suggest that previous coping strategies for managing illness uncertainty had been honed and primed to cope with COVID-19. Perhaps this proactive problem-focused approach reflects the practical skills and responses needed to manage a complex chronic condition which all the participants reported having had to develop, as uncertainty had become embedded in their lives and given them skills to cope with even further uncertainty. Indeed, several strategies were used to manage uncertainty, decided upon after careful consideration and in response to, for example, the level of impairment felt, social support available, and consideration of wider resources. Consistently with this, Lazarus and Folkman's conceptualisation, with illness and the pandemic-related uncertainties affecting firstly primary and then secondary appraisals, was also supported.

### Limitations

This study has a number of limitations. Our sample was purposive, consisting of generally younger and physically healthy PwP with access to the internet, IT skills, and support by spouses/family. Thus, not all PwP may have the same adaptive response to the double threat posed by Parkinson's and COVID-19. Moreover, in order to limit the participants’ burden, no member-check appointments to discuss the data with them were scheduled following the interviews. Therefore, more targeted methods including member-checks and more diverse samples may allow for further in-depth explorations of illness uncertainty in PwP.

### Clinical implications

Despite calls for more research on managing uncertainty,^41,42^ specifically targeted interventions are still scarce. Where these have been developed (e.g. Germino et al.,^43^ Hoff et al.^44^) they often include techniques such as relaxation training and reframing threatening cognitions to ones emphasising opportunity. Healthcare communication – especially framed around hope – is also paramount,^45,46^ and the issue around appointments with clinicians being cancelled during the pandemic is therefore worrying.^28^ A recent review on how nursing support can reduce illness uncertainty indicated that facilitating patients’ journey through the healthcare system (e.g. limiting diagnosis delay or improving the timing of interventions or care adaptations) is essential, especially during healthcare crises.^47^

Mindfulness-based approaches aiming at increasing people's uncertainty tolerance^48^ may be helpful, as provisional evidence has shown these can be help improve general well-being and psychological distress in PwP (see Zarotti et al.^11^ for a recent review). Furthermore, although the reduction in tolerance of uncertainty has not been formally measured as an outcome in mindfulness studies with PwP, it has been reported as an outcome in qualitative evaluations.^49^

Intervention studies have indicated that illness uncertainty can be reduced by many of the techniques implicitly used by participants in this study – for example, emotion regulation and psychoeducation. Other factors are also suitable targets for intervention – for example, social support, illness support, establishing confidence in health care. Discuss coping mechanism is an important part of managing illness uncertainty. Our findings are consistent with those of Mishel (1990), who argued that if the coping strategies are effective, adaptation will occur.

### Conclusions

This study has shed light on the experiences of PwP at a time of an unprecedented international health crisis. Albeit not without significant challenges, participants reported a range of implicit and explicit strategies to cope with the ‘double whammy’ of illness uncertainty. While generally successful in maintaining well-being, it is important that these are used to help inform interventions and strategies for those in different situations and who might need additional support.

## Notes

1. Hereafter called ‘Parkinson's’, the preferred term of the largest UK charity for the condition, Parkinson's UK.
